# Profiling extra cellular matrix associated proteome of human fetal nucleus pulposus in search for regenerative targets

**DOI:** 10.1038/s41598-021-97620-w

**Published:** 2021-09-24

**Authors:** Shanmuganathan Rajasekaran, Chitra Thangavel, Niek Djuric, Muthurajan Raveendran, Dilip Chand Raja Soundararajan, Sharon Miracle Nayagam, Monica Steffi Matchado, K. S. Sri Vijay Anand, Krishna Venkateshwaran

**Affiliations:** 1 Department of Spine Surgery, Ganga Hospital, 313, Mettuppalayam Road, Coimbatore, 641043 India; 2 Ganga Research Centre, No 91, Mettuppalayam Road, Coimbatore, 641030 India; 3 Department of Neurosurgery, Leiden University Medical Center, Leiden, The Netherlands; 4 Department of Plant Biotechnology, Tamil Nadu Agricultural University, Coimbatore, 641003 India; 5 Department of Clinical Pathology, Microlab, Coimbatore, India

**Keywords:** Immunochemistry, Proteomics, Preclinical research

## Abstract

Degeneration of the intervertebral disc is associated with a decrease in extra-cellular matrix (ECM) content due to an imbalance in anabolic and catabolic signaling. Our previous study profiled the core matrisome of fetal NP’s and identified various proteins with anabolic potential for regenerative therapies. This study aims to complement those results by exploring ECM regulators, associated proteins and secreted factors of the fetal nucleus pulposus (NP). Proteomic data of 9 fetal, 7 healthy adults (age 22–79), and 11 degenerated NP’s was analyzed. Based on the selection criteria, a total of 45 proteins were identified, of which 14 were uniquely expressed or upregulated in fetus compared to adult NP’s. Pathway analysis with these proteins revealed a significant upregulation of one pathway and two biological processes, in which 12 proteins were involved. Prolyl 4 hydroxylase (P4HA) 1 and 2, Procollagen-lysine, 2-oxoglutarate 5-dioxygenase (PLOD) 1, and Heat shock protein 47 (SERPINH1) were involved in ‘collagen biosynthesis’ pathway. In addition, PLOD 1, SERPINH1, Annexin A1 and A4, CD109 and Galectin 3 (LGALS3) were all involved in biological process of ‘tissue development’. Furthermore Annexin A1, A4 and A5, LGALS-3 and SERPINF1 were featured in ‘negative regulation of cell death’. In conclusion, additionally to core ECM proteome, this study reveals ECM regulators and ECM affiliated proteins of interest to study for regenerative therapies, and their potential should be validated in future mechanistic experiments.

## Introduction

One of the most debilitating diseases of the twenty-first century is low back pain^1^. It has a lifetime prevalence of 65–80%, and affects 10–30% of the population every year^2^. Even though low back pain can be caused by a great variety of underlying pathologies, most complaints will come from intervertebral disc degeneration (DD). During the degeneration process, the disc loses its protein and water content^3^, which often results in a decreased height and strength and is often accompanied by a chronic inflammatory process^4–6^.

Recent studies have explored the underlying molecular mechanism and proteomic signature of DD^4, 7, 8^. They found that the decrease in height and loss of water could be attributed to a loss of extracellular matrix proteins^4^, and in particular due to a loss in proteoglycans, which are responsible for remaining the hydrostatic pressure of the disc^3^. The loss of these proteins could likely be attributed due to an increase in catabolic pathways combined with a decrease in anabolic pathways^8^. Therefore, the current focus of DD research is finding a regenerative therapy that could stimulate anabolic pathways or inhibit the catabolic ones.

The majority of the research that focuses on regenerative therapies, study degenerated discs in comparison with healthy discs^6–8^. Even though this approach will nicely quantify the differences and reveal areas of special interest. It will not reveal any new proteins with regenerative potential that are seldom present in any of these discs.

In our previous study, we explored the core matrisome (ECM proteome) of human fetal nucleus pulposus (NP) for proteins of regenerative potential and found a great number of such proteins to be upregulated or uniquely expressed in the fetal NP’s^9^**.** Nevertheless, only 86% of the matrisome is core matrisome (Collagens, proteoglycans, and glycoproteins) leaving 14% of the fetal matrisome undiscovered.

Therefore, the aim of this study is to explore the regenerative potential of the fetal ECM regulators, affiliated proteins and secreted factors through comparing its proteomic signature with healthy adult NP’s.

## Materials and methods

### Patient population

Two main groups were compared in this study. Fetal NP tissue was harvested from 9 miscarriages from all lumbar and thoracic spinal segments at a developmental stage of less than 6 months. Healthy adult NP tissues were harvested from brain dead organ donor volunteers, of which 2 were harvested from L3 to L4 and 5 from L4 to L5 discs. For additional analysis, 11 degenerated discs were included, 1 originated from the level L2 to L3, 1 from L3 to L4, 7 from L4 to L5 and 2 from L5 to S1. The extent of degeneration was scored according to Pfirmann classification (Supplementary Table S1). Institutional Review Board (Ganga Medical Centre and Hospitals, Regn No: EC/NEW/INST/2020/1146) approval were obtained to conduct research on these specimens. Written informed consent was obtained, and all methods were carried out according to the Declaration of Helsinki. A detailed description of the sample collection procedure was described earlier by Rajasekaran et al. 2020^9^.

### Sample processing

Around 100 mg from 9 fetuses and 200 mg tissue from 7 adults and 11 degenerated discs were subjected for extraction of total proteins and subjected to ESI–LC–MS/MS with conditions as described in our earlier report: Rajasekaran et al.^10^.

### Bioinformatics analysis

Bioinformatic analysis was performed in the same way as PART 1^11^: MS/MS raw data acquired from Orbitrap Velos Pro Mass Spectrometer were analyzed by Proteome Discoverer v1.4 using Mascot (Matrix Science, London, UK; version 2.4.1.0) and inbuilt SequestHT search algorithm. The peptide spectrum matches (PSMs) from SequestHT and Mascot were post-processed using the Percolator algorithm. The peptides with rank one and having a q-value < 0.01 were considered for protein identification. Gene Ontology and Pathway enrichment analysis were carried out using The Database for annotation, visualization, and integrated discovery (DAVID) version 6.8. STRING database v 10.5 was used for pathway analysis, whereas Cytoscape vs. 3.7.0 was used for protein–protein interaction analysis.

### Relative quantification by spectral count

Spectral counts obtained by LC/MS–MS were further subjected to normalization by normalized spectral abundance factor (NSAF) method as described by Zybailov et al.^12^. NSAF is a relative quantification method based on protein length and spectral count. NSAF is calculated as follows:

$$\left( {NSAF} \right)k = \frac{{\left( \frac{SpC}{L} \right)k}}{{\mathop \int \nolimits_{i = 1}^{N} \left( \frac{SpC}{L} \right)i}}$$

SpC: Number of spectral counts; L: Protein Length; k: individual protein, N: number of all proteins in the experiment.

### Quantitative analysis

Out of the proteomic database, the matrisome associated proteins (regulators, ECM affiliated, and secreted factors), as defined by Naba et al. were selected for further analysis^13^. In addition to this list, the rest of the proteome was screened manually for other not core matrisome proteins present in the ECM. All selected proteins with > 2 unique peptides or 1 unique peptide with a PSM ≥ 10 were included in the analysis^14^, Proteins were only considered as potential marker if they were present in four or more fetus samples.

### Statistical analysis

Data was analyzed using SPSS software version 25. Since the sample size was limited, differences in protein expression between groups were assessed using Mann–Whitney U tests. Statistical analysis was performed if that protein was present in four or more samples in both groups. The two-tailed alfa level was set at 0.05. Samples with missing values were excluded from the analysis.

### Pathway analysis

For the purpose of identifying which fetus-specific proteins of interest might qualify for future regenerative treatment, all fetal-specific proteins were submitted to a pathway enrichment analysis using both STRING and DAVID databases version 6.8 Proteins were regarded as ‘fetus specific’ if they met one of the following three criteria: Firstly, if they were uniquely expressed in fetal NP’s. Secondly, if fetal NP’s showed a significant upregulation compared to healthy adult NP’s, thirdly, if the healthy adult group expressed a protein in less than four samples, an upregulation in fetal NP’s of at least a twofold also qualified.

### Interaction analysis

In order to find out whether the identified pathways interact and share common initiators, a protein–protein interaction analysis was performed using Cytoscape vs 3.7.0.

### Verification in degenerated samples

For further verification of regenerative relevance, proteins that decreased from fetus to healthy adult NP’s and that were identified in regenerative pathways were used in an additional analysis to assess whether a further decrease was seen in severely degenerated discs. For the selected proteins, expression levels from 11 degenerated NP’s were compared to expression levels in fetus and healthy adult NP’s.

### Validation by immunohistochemistry

For validation of proteomic data, immunohistochemistry (IHC) was used. Based on the results of the statistical, pathway, and interaction analysis, two proteins were chosen for validation: SEPRINH1 and ANXA4. 10 fetus and 5 adult NP samples were analysed. Samples were fixed in formalin solution and embedded in paraffin. From the paraffin blocks, 5 μm section were used for IHC using a three-step indirect method. Samples were heated in retrieval buffer (Tris pH 9.5 and Borate pH 8.0) until 15 pounds per square inch pressure, which was thereafter maintained for 2 min. Subsequently, slices were rinsed in ethanol solutions, and incubated in 0.1% hydrogen peroxide to achieve blocking. Monoclonal antibodies for SERPINH1 (Santa Cruz Biotechnology, Inc USA) and ANXA4 (Santa Cruz Biotechnology, Inc USA) were used. Primary antibodies (1:500 dilution) were incubated for 1 h and secondary antibodies (1:1000 dilution) were developed with DAB and counterstained with Harris hematoxylin. Image evaluation was done using a Leica DML light microscope with Leica Application Suite Vs 4.5.0.418 software. Positive slides were scored as a mild(+)/moderate(++)/strong(+++) and location of the staining was taken into account. If staining was absent, slides were scored as negative.

## Results

### Descriptive analysis of ECM

**Figure 1:**
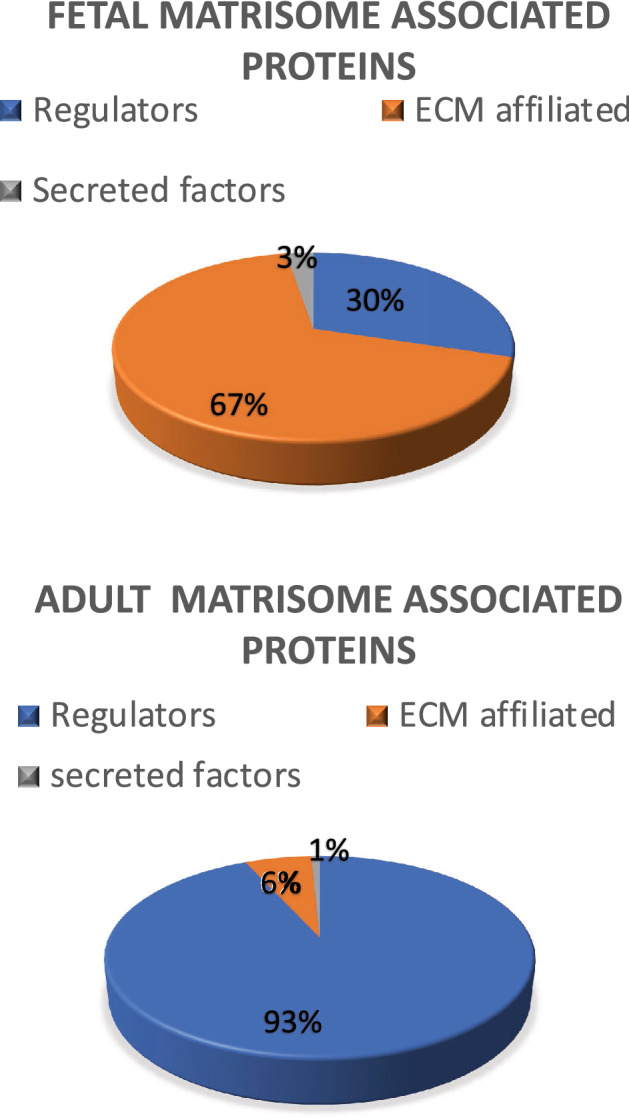
Relative protein distribution. Non-core matrisome composition of fetus and healthy adult NP’s.

The general proteomic features of the discs were described earlier by Rajasekaran et al.^9^. The matrisome consisted for 14% of matrisome associated proteins in fetal NP’s; similar results were seen in adult discs (10%)^11^. These proteins were subdivided into regulators (30% in fetus and 93% in adult), ECM affiliated proteins (67% in fetus and 6% in adult), and secreted products (3% in fetus and 1% in adult). The distribution of matrisome associated proteins is illustrated in Fig. 1. A list of all proteins and the frequency of their expression in fetus and healthy adult NP’s can be found in Supplementary Table S2.

### Comparing fetal and adult ECM

**Figure 2:**
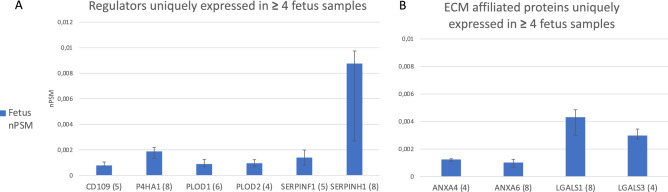
Uniquely expressed proteins. Proteins that were uniquely expressed in fetal NP’s. The Y-axis displays median normalized PSM’s per group, X-axis shows protein gene symbols and the (number of samples) expressing the protein. (**A**) Displays regulators, (**B**) ECM affiliated proteins.

Based on the selection criteria, the quantitative analysis revealed a total of fourteen proteins expressed in ≥ 4 fetus samples, of which ten were uniquely expressed in fetus. Of these ten proteins, six were regulators and four ECM-affiliated proteins (Fig. 2).

**Figure 3:**
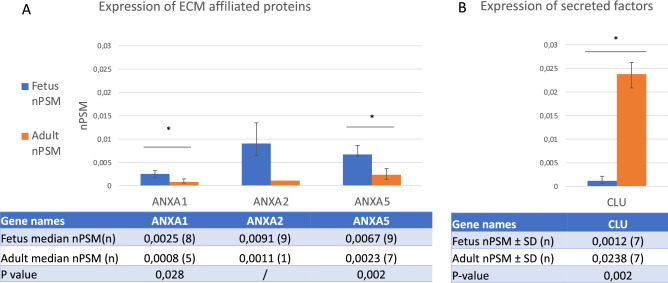
Comparing matrisome associated protein expression. Figure displays the comparisons of expressed proteins between fetus and healthy adults. (**A**) Shows the expression of matrisome affiliated and (**B**) secreted factors. The Y-axis displays median normalized PSM’s per group, X-axis shows protein gene symbols, error bars are Interquartile ranges and * indicates statistical significance. The attached table contains the median (number of samples) for each group and provides the according Mann Whitney U *p* values if statistics could be performed (n ≥ 4). ‘/’ indicates that 3 or less samples were present in one of the groups and no statistical test could be performed.

Moreover, only 3 proteins were expressed in four or more fetal and adult discs simultaneously and thus qualified for statistical analysis. The analysis revealed a significant upregulation of Annexin 1 (*p* = 0.028), and Annexin 5 (*p* = 0.002) in fetal NP’s in comparison with adult NP’s (Fig. 3A). In contrast, a significant upregulation in healthy adult NP’s for secreted factor Clusterin (*p* = 0.002) as compared to fetal NP’s (Fig. 3B).

In addition, Annexin 2 was present in less than four samples in the healthy adult group, which made it unsuitable for statistical analysis. Nevertheless, since Annexin 2 was > twofold upregulated in fetal NP’s, it was incorporated in the pathway analysis (Fig. 3A).

### Pathway analysis for regenerative potential

Based on the exclusion criteria described in the method section. Twelve fetal proteins of interest were selected for anabolic pathways and biological processes (Table 1). The analysis revealed four pathways to be significantly upregulated, of which one was regarded anabolic, and 73 biological processes to be significantly upregulated, out of which two were regarded as anabolic. In these pathways and processes, ten of the twelve proteins were involved (Table 2).

**Table 1:** Protein list of interest.

Protein name	Gene symbol	Cluster
CD109 molecule	CD109	Regulator
Prolyl 4-hydroxylase, alpha polypeptide I	P4HA1	Regulator
Procollagen-lysine 1, 2-oxoglutarate 5-dioxygenase 1	PLOD1	Regulator
Procollagen-lysine 1, 2-oxoglutarate 5-dioxygenase 2	PLOD2	Regulator
Serpin peptidase inhibitor, clade F (alpha-2 antiplasmin, pigment epithelium-derived factor), member 1	SERPINF1	Regulator
Serpin peptidase inhibitor, clade H (heat shock protein 47), member 1, (collagen-binding protein 1)	SERPINH1	Regulator
Annexin A1	ANXA1	ECM affiliated
Annexin A2	ANXA2	ECM affiliated
Annexin A4	ANXA4	ECM affiliated
Annexin A5	ANXA5	ECM affiliated
Annexin A6	ANXA6	ECM affiliated
Lectin, galactoside-binding, soluble, 1	LGALS1	ECM affiliated
Lectin, galactoside-binding, soluble, 3	LGALS3	ECM affiliated

Table lists the proteins of interest (uniquely expressed in fetus in at least 4 samples, significantly upregulated in fetus or upregulated > twofold if only 1 sample expressed the respective protein in the healthy adult group).

**Table 2:** Pathway analysis.

Pathway	Proteins involved	*p* value	Gene symbol
Collagen biosynthesis and modifying enzymes	4	0.0004	P4HA1, SERPINH1, PLOD1,PLOD2
**Biological process**			
Tissue development	6	0.0097	ANXA1,ANXA4,PLOD1,LGALS3,CD109,SERPINH1
Negative regulation of cell death	5	0.0097	ANXA1,ANXA4,ANXA5,LGALS3,SERPINF1

Table displays the results of the STRING and DAVID pathway analysis version 6.8.

The first (left) column shows the identified pathways that are involved in ECM assembly; the second column shows the number of proteins of interest involved in that pathway, the third lists the *p* values of pathways and the fourth displays the gene symbols of the proteins involved.

In total, 10 proteins were identified in one pathway and two biological processes.

The pathway involved in collagen biosynthesis and featured ‘Prolyl 4-hydroxylase, alpha polypeptide 1’ (P4HA1), ‘Procollagen-lysine 1, 2-oxoglutarate 5-dioxygenase’ (PLOD) 1 and 2, and ‘Heat shock protein 47’ (SERPINH1) (Table 2). Moreover, the first biological process identified was ‘tissue development’, which featured Annexin 1 and 4, CD109, Galectin-3 (LGALS3), PLOD1, and SERPINH1. The second subgroup of biological processes was negative regulation of cell death, featuring ANXA1, 4, and 5, LGALS1 and 3, and SERPINF1 (Table 2).

### Interaction analysis

The interaction analysis in Cytoscape revealed that eleven of the twelve proteins were interconnected (Figure S1). CD109 was the only protein which had no interaction. Within the interconnected proteins, two clusters were identified: In the first cluster, ANXA1 was identified as initiator that sends outgoing signals to other ANXA proteins, which in turn transduced their signals to LGALS1,LGALS3 and SERPINH1. In the second cluster, P4HA1 sends outgoing signals to PLOD1 and PLOD2, the latter of which sends signals to SERPINF1 and SERPINH1. Thereby making SEPRINH1 a crucial protein where multiple signal cascades come together (Supplementary Figure 1).

### Verification in degenerated samples

**Figure 4:**
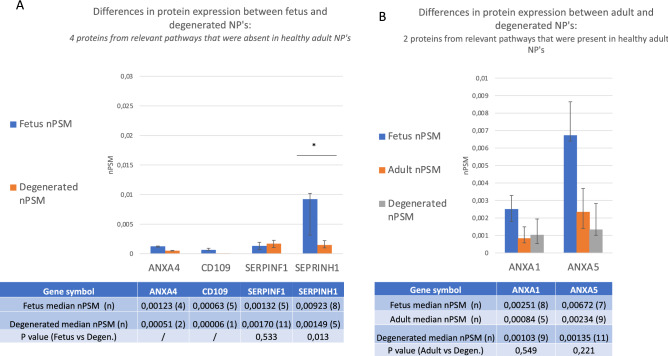
Additional comparison with degenerated samples for the proteins significantly upregulated in anabolic pathways. (**A**) Includes the proteins that were absent in healthy adult NP’s, and shows differences in protein expression between fetus and degenerated discs. (**B**) Includes the proteins that were expressed in a significantly lower amount in healthy adult NP’s compared to fetus and shows the differences in protein expression between healthy adult and degenerated discs. The Y axis displays median normalized PSM’s per group, X axis shows protein gene symbols, error bars are Interquartile ranges and * indicates statistical significance. The attached table contains the median (number of samples) for each group, and provides the according Mann Whitney U *p* values if statistics could be performed (n ≥ 4). ‘/’ indicates that 3 or less samples were present in one of the groups and no statistical test could be performed.

The ten proteins selected in significantly upregulated anabolic regenerative pathways were incorporated into additional analyses. Of these ten upregulated proteins, eight were expressed solely in fetus samples. Four out of these eight proteins were not only absent in healthy adults but also in degenerated NP’s: LGALS3, P4HA1, PLOD1, and PLOD2. However, the other four proteins that were absent in healthy adult NP’s were present in degenerated NP’s: ANXA4, CD109, SERPINH1 showed lesser expression compared to fetus, but SERPINF1 showed similar expression (Fig. 4A). Regarding the two proteins that were present in both fetus and healthy adult, expression levels both ANXA1 and ANXA5 were comparable in degenerated and healthy adult NP’s (Fig. 4B). In short, of the ten proteins of interest that decreased from fetus to healthy adult, four remained absent in degenerated samples, two did not decrease further and three proteins increased slightly and one increased considerably upon degeneration.

### Validation by immunohistochemistry

**Figure 5:**
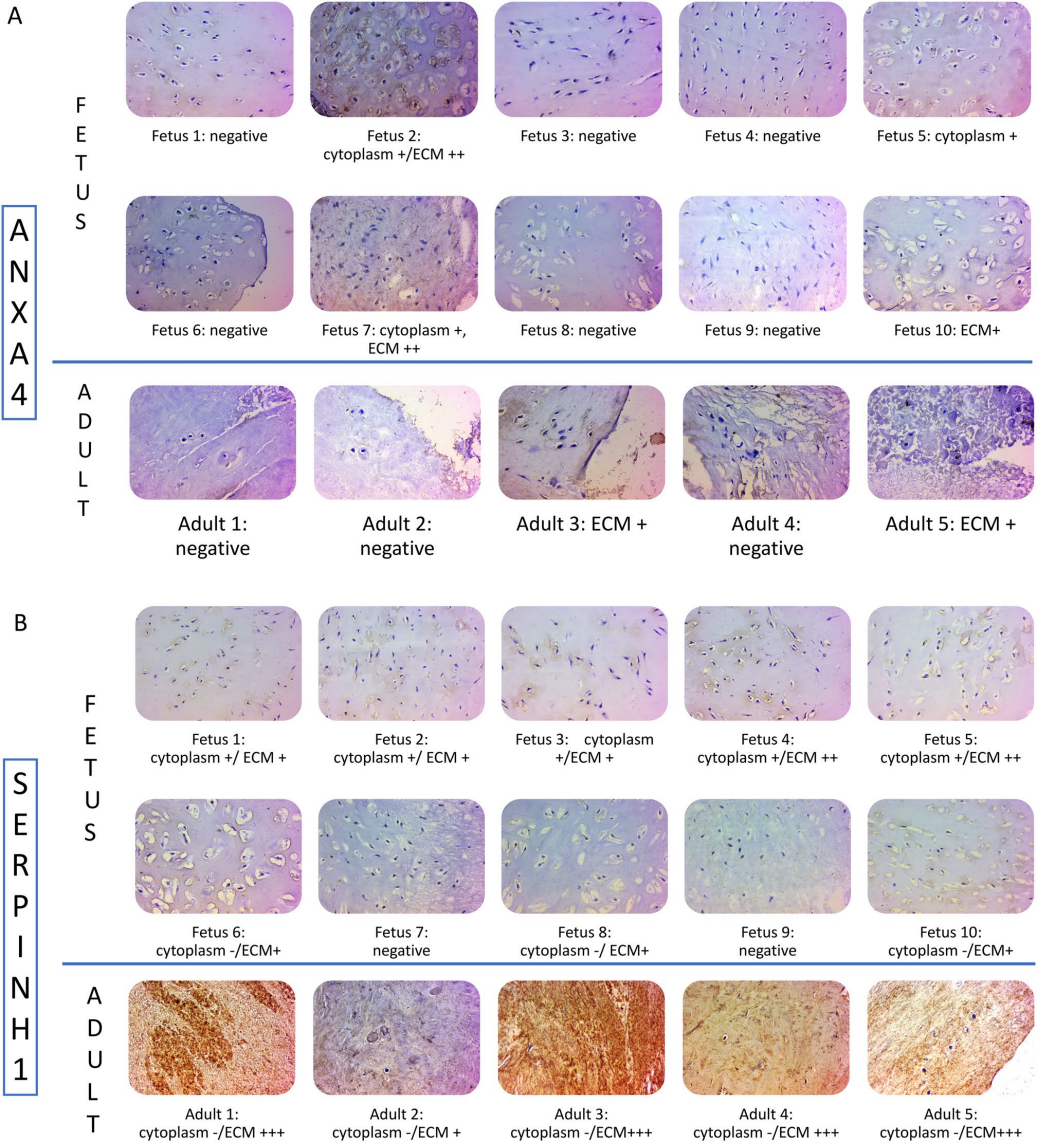
Validation by immunohistochemistry. Immunohistochemistry was used to validate protein expression of SEPRINH1 in (**A**) and ANXA4 in (**B**) (magnification 400x, scale bar = 50 µm). Positive samples are stained brown. Both location and intensity are stated under the pictures: + indicates mild staining, ++ moderate and +++ strong staining.

Validation analysis by IHC was performed on ten fetus and five adult control samples. The analysis validated protein expression of SERPINH1 and ANXA4. Regarding SERPINH1, eight of the ten fetus samples showed mild cytoplasmic and ECM staining. By contrast, adult samples showed strong positive staining, but only of ECM (Fig. 5A). ANXA4 showed moderately positive cytoplasmic and ECM staining in four out of the ten fetus samples and showed mildly positive ECM staining in two out of the 5 adult samples (Fig. 5B).

Taken together, the location of SEPRINH1 differed in fetus and adult, since intracellular staining cannot be quantified in the same way as ECM staining, no conclusion could be drawn on the quantitative difference between fetus and adult. Further, since fetus showed both in and extra cellular ANXA4 reactivity and adult only intracellular, fetal ANXA4 expression was regarded as higher, which was in line with the proteomic results.

## Discussion

In addition to our previous study on core matrisomal proteins in fetal NP’s. This study profiled the ECM regulators, affiliated proteins and secreted factors. A total of ten upregulated proteins of interest were identified in anabolic pathways and biological processes. Moreover, this study was the first to characterize the matrisome associated proteins of human fetal NP’s.

While previous studies have focussed on studying core matrisome proteins of the human fetal disc^15^, no studies have been conducted on the regulators, affiliated proteins, and secreted factors. Nevertheless, a comparable study on bovine disc by Caldeira et al.^16^, reported similar difference between fetuses and adults regarding ANXA2. In contrast, the authors found ANXA 1,4 and 5 to be highly expressed in healthy adults, while this study found higher levels in fetal NP’s. In order to identify whether this is due to a methodological error or an interspecies difference, more proteomic studies on fetal discs should be conducted.

### Distribution differences between healthy adult and fetus discs

Interestingly, fetal discs have a considerably higher percentage of ECM affiliated proteins (67% vs 6%), which is compensated with a lower percentage of Regulators (30% vs 93%). Currently, the reason for this difference remains unknown, and since this is the first study on fetal matrisome associated proteins, no earlier data for comparison is available. Nevertheless, since the extraction of the tissue was performed in a standardized manner without any issues, it seems more likely that the cause for this difference has a biological origin. For example, Annexins, which are highly expressed in fetus, play an important function in endo and exocytosis^17, 18^. One may speculate that in fetal discs where cells are abundant, but ECM yet has to be formed, the need for protein trafficking is higher as compared to healthy adult disc, where the ECM is already build. Hence higher levels of ECM-affiliated proteins such as annexins are needed in fetal NP’s.

### Protein differences between healthy and degenerated adult discs

In the additional analysis with degenerated discs, LGALS3, P4HA1, and PLOD 1 and 2 remained absent in degenerated discs, which further emphasized their relevance as a potential target for regenerative treatments. However, the proteins that increased in degenerated discs should not be regarded as irrelevant. Depending on their specific function, the upregulation in degenerated discs could mean that the respective protein promotes degeneration, but it could also be a compensatory mechanism, in which case the protein expression should be further stimulated in order to combat degeneration. Hence the regenerative potential of the five proteins that increased (ANXA4, CD109, SERPINH1, SERPINF1), should still be closely evaluated in future mechanical experiments. Below, we shall evaluate the available literature on the identified proteins and discuss their relevance based on their functions.

### Anabolic pathways

The pathway analysis revealed four proteins involved in collagen biosynthesis. Out of these four proteins, P4HA1 was involved in the synthesis of Collagen type 1 ,3 and 4, impact on the synthesis of other type of collagens yet has to be confirmed^19^. Interestingly, they showed to be of additional benefit by controlling gene expression of Hypoxia inducible factor, which are essential for the homeostasis of reactive oxygen species^20, 21^. Furthermore, PLOD 1 and 2 are essential for the collagen crosslinking and glycosylation^22^. Unfortunately, the additional regulatory roles of these enzymes in disc disease remain unknown. The last protein involved in this pathway is SERPINH1, a collagen specific heat shock protein involved in stabilizing the collagen triple helix^23^. Taken together, PLOD 1,2, and SERPINH1 present themselves as potential candidates for a collagen biosynthesis promoting therapies in DD. Interestingly, the collagen biosynthesis pathway also stood out in our previous paper on core matrisome proteins with eleven core matrisomal proteins involved. This illustrates that many proteins of the fetal NP’s play a role in collagen synthesis and regulation, highlighting the importance of this pathway for a healthy ECM.

### Tissue development

Eight proteins of interest were involved in the biological process ‘tissue development’. Out of these proteins, the functions of PLOD 1 and 2 and SERPINH1 have already been discussed under collagen biosynthesis. Out of the remaining seven, Annexin A1 is involved in wound healing. However, it also reduces fibrosis through increasing MMP-1, which degrades ECM content^24^. Because of its catabolic potential, Annexin A1 seemed less suitable for regenerative therapies. Annexin A4 is an immune regulator that is also involved in tubular development^25^, but whether it also contributes to ECM development in the NP remains to be elucidated. In addition, CD109 is involved as a regulator of the transforming growth factor-beta, a protein that is associated with regeneration of IVD’s^26^. CD109 functioning as a TGF-beta co-receptor when attached to a membrane, but as an antagonist in a soluble form^27^. Since it will likely be soluble in a regenerative therapy, CD109 will inhibit growth and tissue development rather than induce it. This makes CD109 ineffective for regenerative therapies. It should be noted that the presence of CD109 could potentially be explained by contamination with blood cells, but since it was absent in healthy NP’s and only present in marginal quantities in degenerated NP’s, this seems less likely.

### Negative regulation of cell death

Five proteins were involved in the negative regulation of cell death, out of which ANXA1 was regarded unsuitable because of its catabolic effect through MMP-1 as described under ‘tissue development^24^. ANXA4 was deemed to be of a higher potential: it negatively regulates apoptotic signaling and decreases the catabolic effect of inflammation through inhibition of NF-kB^28, 29^. ANXA5 was involved in both positive and negative regulation of apoptotic processes^28, 30^. Unfortunately, only little is known regarding its functions. Therefore, no conclusions can be drawn regarding its regenerative potential. Further, Galactin-3 (LGALS3) has shown to be an inhibitors of the extrinsic apoptotic pathway in multiple cell types^31^. At last, SERPINF1 was associated with negative regulation of cell death in neurons, but whether this is translatable to NP cells remains unknown.

When integrating the relevance of all proteins in the selected pathways, only three out of the ten proteins remain interesting for regenerative therapies, all of which are regulators: PLOD 1 and 2 due to their involvement in collagen crosslinking and glycosylation, SERPINH1 for stabilizing the collagen triple helix. In addition, three proteins have shown potential in other tissues, but their effects on ECM remain unknown: the regulator SERPINF1 for its negative regulation of endopeptidases and possibly negative regulation of cell death, the ECM affiliated protein ANXA4 due to its inhibition of catabolic processes and ECM affiliated protein LGALS3 for inhibiting apoptosis. Out of the 6 abovementioned proteins, the interaction analysis showed that SERPINH1 would be the most interesting targets for treatment options. Beside, this protein was also upregulated in degenerated samples compared to healthy adult samples, possibly as a compensatory mechanism to combat degeneration.

### Limitations

This study has several strong points: This study was the first to report on matrisome associated proteome in human fetal discs. Nevertheless, this study also has some limitations. For instance, due to the low sample size and explorative nature of the study, a decision was made not to correct for multiple testing. Furthermore, the protein detection threshold of this study was limited by the sensitivity of mass spectrometer (q-value < 0.01), inferring that potentially interesting proteins with lower expression values might have been overlooked. At last, this study may also have overlooked potentially interesting intracellular proteins with relevant extracellular effects that were not characterized as matrisome associated.

In short, we have demonstrated that many fetus specific matrisomal associated proteins exist and that a considerable part of them are involved in anabolic pathways, which suggests that they may be interesting targets for developing regenerative therapies for disc degeneration and/or aging. Nevertheless, only little is known regarding the molecular pathways in the NP of most of proteins identified. This highlight that the great variety of possibilities for regenerative medicine is poorly understood, which indicate that more molecular research on the NP proteome is needed. Moreover, future studies should focus on verifying the regenerative potential of the proposed proteins in mechanical experiments, in which an abundance of one of these proteins is added to adult discs to see if degeneration can be prevented, or to degenerated discs, to see if degeneration can be reversed.

## Supplementary Information

Supplementary Information.

## References

[1] Collaborators G.D.A.I.I.A.P (2016). Global, regional, and national incidence, prevalence, and years lived with disability for 310 diseases and injuries, 1990–2015: A systematic analysis for the Global Burden of Disease Study 2015. Lancet.

[2] Urits I (2019). Low back pain, a comprehensive review: Pathophysiology, diagnosis, and treatment. Curr. Pain Headache Rep..

[3] Roughley PJ, Alini M, Antoniou J (2002). The role of proteoglycans in aging, degeneration and repair of the intervertebral disc. Biochem. Soc. Trans..

[4] Roughley, P.J., *Biology of intervertebral disc aging and degeneration: involvement of the extracellular matrix.* Spine (Phila Pa 1976), 2004. **29**(23): p. 2691–9. 10.1097/01.brs.0000146101.53784.b1 15564918

[5] Feng H (2006). Extracellular matrix in disc degeneration. J. Bone Jt. Surg. Am..

[6] Vo NV (2016). Molecular mechanisms of biological aging in intervertebral discs. J. Orthop. Res..

[7] Yee A (2016). Fibrotic-like changes in degenerate human intervertebral discs revealed by quantitative proteomic analysis. Osteoarthr. Cartil..

[8] Ding F, Shao ZW, Xiong LM (2013). Cell death in intervertebral disc degeneration. Apoptosis.

[9] Rajasekaran S (2020). Part 1: Profiling extra cellular matrix core proteome of human fetal nucleus pulposus in search for regenerative targets. Sci. Rep..

[10] Rajasekaran S (2017). ISSLS PRIZE IN CLINICAL SCIENCE 2017: Is infection the possible initiator of disc disease? An insight from proteomic analysis. Eur. Spine J..

[11] Rajasekaran S, Tangavel C, Djuric N, Raveendran M, Soundararajan DC, Nagayam SM, Matchado MS, Anand KS (2020). Part 1: Profiling extra cellular matrix core proteome of human fetal nucleus pulposus in search for regenerative targets. Sci. Rep..

[12] Zybailov B (2006). Statistical analysis of membrane proteome expression changes in *Saccharomyces cerevisiae*. J. Proteome Res..

[13] Naba A (2016). The extracellular matrix: Tools and insights for the “omics” era. Matrix Biol..

[14] Higdon R, Kolker E (2007). A predictive model for identifying proteins by a single peptide match. Bioinformatics.

[15] Smith SM (2009). Topographical variation in the distributions of versican, aggrecan and perlecan in the foetal human spine reflects their diverse functional roles in spinal development. Histochem. Cell Biol..

[16] Caldeira J (2017). Matrisome profiling during intervertebral disc development and ageing. Sci. Rep..

[17] Konopka-Postupolska D, Clark G (2017). Annexins as overlooked regulators of membrane trafficking in plant cells. Int. J. Mol. Sci..

[18] Rentero C (2018). Annexins—Coordinators of cholesterol homeostasis in endocytic pathways. Int. J. Mol. Sci..

[19] Xiong G (2014). Prolyl-4-hydroxylase alpha subunit 2 promotes breast cancer progression and metastasis by regulating collagen deposition. BMC Cancer.

[20] Fujita N (2012). Expression of prolyl hydroxylases (PHDs) is selectively controlled by HIF-1 and HIF-2 proteins in nucleus pulposus cells of the intervertebral disc: Distinct roles of PHD2 and PHD3 proteins in controlling HIF-1alpha activity in hypoxia. J. Biol. Chem..

[21] Scortegagna M (2003). Multiple organ pathology, metabolic abnormalities and impaired homeostasis of reactive oxygen species in Epas1^–/–^ mice. Nat. Genet..

[22] Qi Y, Xu R (2018). Roles of PLODs in collagen synthesis and cancer progression. Front. Cell Dev. Biol..

[23] Ono T (2012). Direct in vitro and in vivo evidence for interaction between Hsp47 protein and collagen triple helix. J. Biol. Chem..

[24] Alam A (2014). Redox signaling regulates commensal-mediated mucosal homeostasis and restitution and requires formyl peptide receptor 1. Mucosal Immunol..

[25] Seville RA (2002). Annexin IV (Xanx-4) has a functional role in the formation of pronephric tubules. Development.

[26] Abbott RD (2012). Regenerative potential of TGFbeta3+ Dex and notochordal cell conditioned media on degenerated human intervertebral disc cells. J. Orthop. Res..

[27] Litvinov IV (2011). CD109 release from the cell surface in human keratinocytes regulates TGF-beta receptor expression, TGF-beta signalling and STAT3 activation: Relevance to psoriasis. Exp. Dermatol..

[28] Bruneel A (2005). Proteomics of human umbilical vein endothelial cells applied to etoposide-induced apoptosis. Proteomics.

[29] Jeon YJ (2010). Annexin A4 interacts with the NF-kappaB p50 subunit and modulates NF-kappaB transcriptional activity in a Ca^2+^-dependent manner. Cell. Mol. Life Sci..

[30] Gaudet P (2011). Phylogenetic-based propagation of functional annotations within the Gene Ontology consortium. Brief. Bioinform..

[31] Hu Z (2012). Downregulation of galectin-3 by EGF mediates the apoptosis of HepG2 cells. Mol. Cell. Biochem..

