# Supplementary material for: Profiling extra cellular matrix associated proteome of human fetal nucleus pulposus in search for regenerative targets
Source: Sci Rep. 2021 Sep 24;11:19013. doi: 10.1038/s41598-021-97620-w (PMC8463528; doi:10.1038/s41598-021-97620-w)

**Profiling extra cellular matrix associated proteome of human fetal nucleus pulposus in search for regenerative targets**

Shanmuganathan Rajasekaran Prof. MD.^1^, Chitra Thangavel PhD.^2^, Niek Djuric^2,3^, Muthurajan Raveendran Prof.^4^, Dilip Chand Raja Soundararajan ^1^, Sharon Miracle Nayagam^2^, Monica Steffi Matchado^2^, K S Sri Vijay Anand^1^, Krishna Venkateshwaran MD^5^.

1. Department of Spine Surgery, Ganga Hospital, 313, Mettuppalayam Road, Coimbatore, 641043, India.

2. Ganga Research Centre, No 91, Mettuppalayam Road, Coimbatore, 641030, India.

3. Department of Neurosurgery, Leiden University Medical Center, Leiden

4. Department of Plant Biotechnology, Tamil Nadu Agricultural University, Coimbatore, 641003, India.

5. Department of Clinical Pathology, Microlab Coimbatore, India.

**Corresponding author:**

Shanmuganathan Rajasekaran, Department of Spine Surgery, Ganga Hospital, 313, Mettuppalayam Road, Coimbatore, 641043, India. 91-422-2485000, rajasekaran.orth@gmail.com

**Supplementary appendix**

Table S1

**Pfirrmann grade**Table S1 lists the degeneration scores (Pfirrmann grade) for all proteomic samples.

| **Sample number** | **Fetus** | **Healthy adult** | **Degenerated** |
| --- | --- | --- | --- |
| 1 | 1 | 3 | 4 |
| 2 | 1 | 2 | 5 |
| 3 | 1 | 2 | 5 |
| 4 | 1 | 2 | 4 |
| 5 | 1 | 2 | 4 |
| 6 | 1 | 2 | 5 |
| 7 | 1 | 2 | 4 |
| 8 | 1 |  | 4 |
| 9 | 1 |  | 4 |
| 10 |  |  | 5 |
| 11 |  |  | 5 |

Table S2 **Protein list and expression frequency**

Table S1 lists all matrisome associated proteins found in fetus and/or healthy adult NP’s. the numbers in the most right colums indicates the number of samples in that group that expressed the respective proteins. The total number of samples was 9 for fetus and 7 for healthy adults. Missing values were excluded from the analysis.

| **Protein name** | **Gene symbol** | **Cluster** | **fetus** | **normal** |
| --- | --- | --- | --- | --- |
| Alpha-2-macroglobulin | A2M | Regulator | 1 | 7 |
| Angiotensinogen (serpin peptidase inhibitor, clade A, member 8) | AGT | Regulator | 0 | 3 |
| CD109 molecule | CD109 | Regulator | 5 | 0 |
| Coagulation factor XIII, A1 polypeptide | F13A1 | Regulator | 0 | 5 |
| HtrA serine peptidase 1 | HTRA1 | Regulator | 0 | 7 |
| inter-alpha (globulin) inhibitor H2 | ITIH2 | Regulator | 1 | 0 |
| Prolyl 4-hydroxylase, alpha polypeptide I | P4HA1 | Regulator | 7 | 0 |
| Prolyl 4-hydroxylase, alpha polypeptide II | P4HA2 | Regulator | 3 | 0 |
| Procollagen-lysine 1, 2-oxoglutarate 5-dioxygenase 1 | PLOD1 | Regulator | 6 | 0 |
| Procollagen-lysine, 2-oxoglutarate 5-dioxygenase 2 | PLOD2 | Regulator | 4 | 0 |
| Procollagen-lysine, 2-oxoglutarate 5-dioxygenase 3 | PLOD3 | Regulator | 2 | 0 |
| Alpha-1-antitrypsin | SERPINA1 | Regulator | 2 | 5 |
| Alpha-1-antichymotrypsin | SERPINA3 | Regulator | 1 | 7 |
| Plasma serine protease inhibitor | SERPINA5 | Regulator | 0 | 7 |
| Serpin B12 | SERPINB12 | Regulator | 1 | 0 |
| Glia-derived nexin | SERPINE2 | Regulator | 3 | 7 |
| Pigment epithelium-derived factor | SERPINF1 | Regulator | 5 | 0 |
| Plasma protease C1 inhibitor | SERPING1 | Regulator | 0 | 7 |
| Serpin H1 | SERPINH1 | Regulator | 8 | 0 |
| Transglutaminase 2 | TGM2 | Regulator | 1 | 0 |
| Transglutaminase 3 | TGM3 | Regulator | 1 | 0 |
| Metallopeptidase inhibitor 1 | TIMP1 | Regulator | 0 | 1 |
| Metallopeptidase inhibitor 3 | TIMP3 | Regulator | 0 | 5 |
| Annexin A1 | ANXA1 | ECM affiliated | 8 | 5 |
| Annexin A2 | ANXA2 | ECM affiliated | 9 | 1 |
| Annexin A4 | ANXA4 | ECM affiliated | 4 | 0 |
| Annexin A5 | ANXA5 | ECM affiliated | 9 | 7 |
| Annexin A6 | ANXA6 | ECM affiliated | 8 | 0 |
| C-type lectin domain family 3, member A | CLEC3A | ECM affiliated | 3 | 4 |
| C-type lectin domain family 3, member B | CLEC3B | ECM affiliated | 3 | 0 |
| C-type lectin domain family 11, member A | CLEC11A | ECM affiliated | 2 | 0 |
| Hemopexin | HPX | ECM affiliated | 1 | 0 |
| Lectin, galactoside-binding, soluble, 1 | LGALS1 | ECM affiliated | 8 | 0 |
| Lectin, galactoside-binding, soluble, 3 | LGALS3 | ECM affiliated | 4 | 0 |
| Fibroblast growth factor binding protein 2 | FGFBP2 | Secreted factors | 0 | 1 |
| Filaggrin | FLG | Secreted factors | 1 | 0 |
| Filaggrin family member 2 | FLG2 | Secreted factors | 0 | 1 |
| Frizzled-related protein | FRZB | Secreted factors | 1 | 1 |
| Hornerin | HRNR | Secreted factors | 1 | 0 |
| S100 calcium binding protein A1 | S100A1 | Secreted factors | 1 | 0 |
| S100 calcium binding protein A4 | S100A4 | Secreted factors | 1 | 0 |
| S100 calcium binding protein A8 | S100A8 | Secreted factors | 1 | 1 |
| S100 calcium binding protein A9 | S100A9 | Secreted factors | 0 | 1 |
| S100 calcium binding protein A10 | S100A10 | Secreted factors | 2 | 0 |

**Supplementary figure S1
Interaction analysis**

Figure 4 displays the Interaction analysis of all the proteins of interest, 11 of the 12 proteins were interconnected. Cluster 1 starts with outgoing signals from ANXA1, whereas cluster 2 starts with outgoing signals from P4Ha1, both clusters come together with incoming signals in SERPINH1.


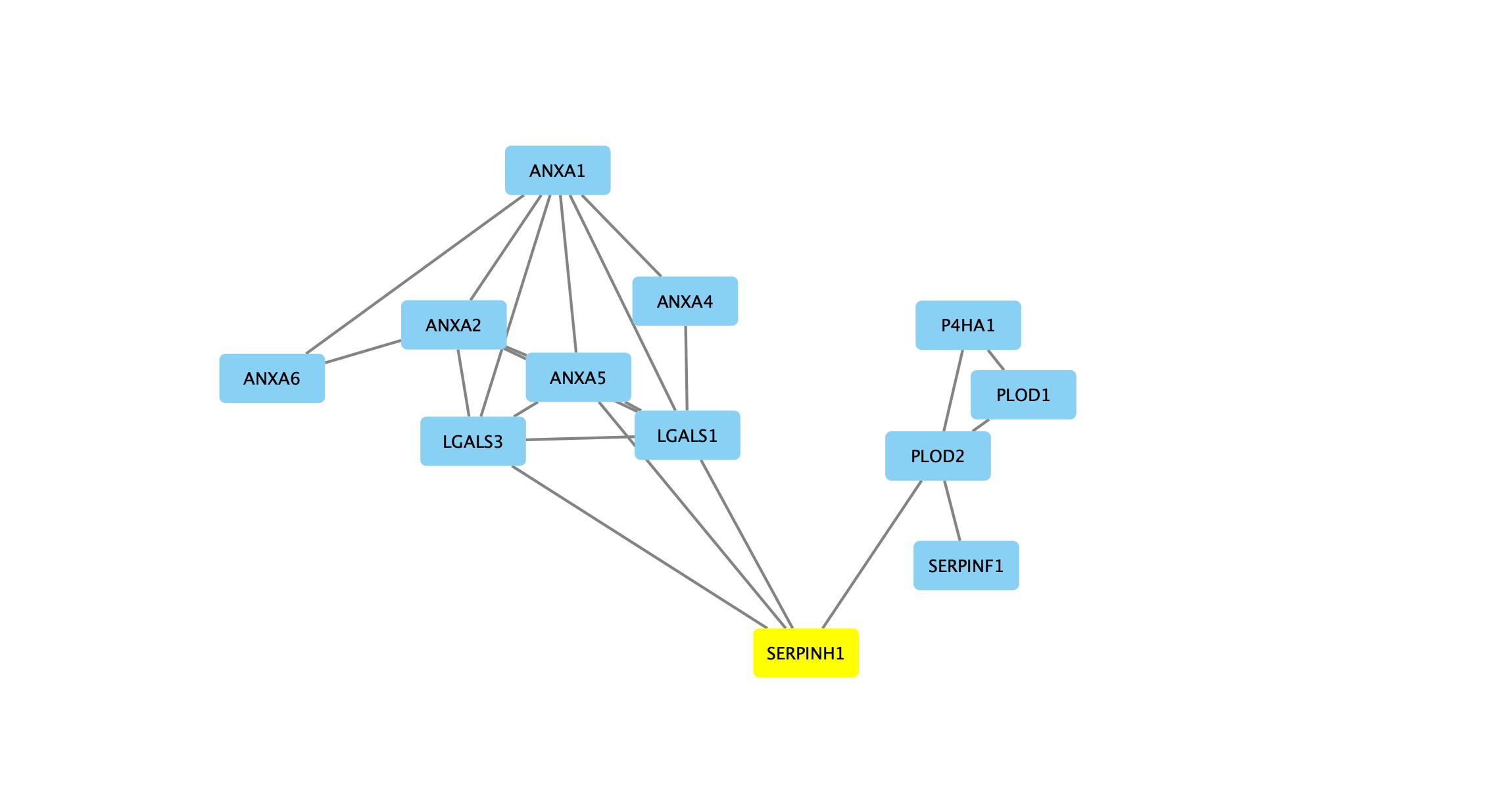

Supplement: Supplementary file 1 — Supplementary Information. [file 41598_2021_97620_MOESM1_ESM.docx]
